# Inhibitor of Tec kinase, LFM-A13, decreases pro-inflammatory mediators production in LPS-stimulated RAW264.7 macrophages via NF-κB pathway

**DOI:** 10.18632/oncotarget.16212

**Published:** 2017-03-15

**Authors:** Fei Wang, Wei Zhang, Chao Wang, Xu Fang, Hao Cheng, Sheng Liu, Xu-Lin Chen

**Affiliations:** ^1^ Department of Burns, The First Affiliated Hospital of Anhui Medical University, Hefei, Anhui, PR China; ^2^ Department of Nephrology, The First Affiliated Hospital of Anhui Medical University, Hefei, Anhui, PR China

**Keywords:** lipopolysaccharide, Tec kinase, NF-κB, macrophages, Immunology and Microbiology Section, Immune response, Immunity

## Abstract

Tec kinase, a prototypical member of the Tec tyrosine kinases family, was shown to mainly govern lymphocyte proliferation. In the present study, we investigated the role of Tec kinase in acute inflammatory response in lipopolysaccharide (LPS) challenge. First, we demonstrate that Tec kinase activity was observed in RAW264.7 macrophages exposed to LPS. Tec and phosphorylated Tec expression were upregulated in a dose- and time-dependent manner after LPS stimulation. LPS increased monocyte chemotactic protein (MCP)-1 secretion and intercellular adhesion molecule (ICAM)-1 expression, and increasing mRNA expression was consistently observed. LPS also induced IκBα phoshporylaytion and its degradation, increased NF-κB p65 phoshporylaytion and translocation to nuclei in RAW264.7 cells. Pretreatment with LFM-A13 decreased LPS-induced cytokines and chemokines production and mRNA levels, blocked NF-κB transactivation. These effects of LPS were also prevented by Tec-siRNA. Additionally, LFM-A13 or Tec-siRNA obviously inhibited LPS-induced TGFβ-activated kinase 1(TAK1) phosphorylation. Taken together, our results suggest that Tec kinase involves in acute inflammation process in LPS-stimulated RAW264.7 cells, at least mediated by activating TAK1/ NF-κB signal pathway.

## INTRODUCTION

Innate immune cells such as macrophages and neutrophils constitute a front line of defense against most microbial infection and are responsible for invading pathogens. In response to bacterial toxins, various inflammatory mediators individually or in combination may dysregulate the immune response and promote tissue-damaging inflammation, contributing to the pathogenesis of lethal LPS challenge [1, 2].

Tec kinases, non-receptor tyrosine kinase family, consist of five members: Tec, Btk, ITK/Emt/Tsk, Bmx and Txk/Rlk [3]. They constitute the second largest family of cytoplasmic tyrosine kinases in humans. Tec kinase is initially discovered in hepatocellular carcinoma, and subsequently found in other cell lineages such as hematopoietic cells, liver and kidney cells [4, 5]. Tec protein was also abundantly expressed in mouse cardiac myocytes after ischemia-reperfusion [6]. In human neutrophils, Tec kinase is suppressed by Insulin-like growth factor (IGF)-1 and augments cell survival by inhibition of necrosis [7]. Besides regulation of proliferation, differentiation and cell survival, Tec kinase families are involved in the regulation of cytokines expression. In neutrophils, Tec and Btk kinases were shown to be expressed and functional, activated by the chemoattractant fMLP [8]. Furthermore, Gilbert C et al. showed that Tec kinases increased fMLP-induced superoxide production, adhesion, and chemotaxis through p38 MAPK in human neutrophils [9].

Besides the neutrophils, the macrophage is the pivotal effector cell in inflammatory reaction in inflammatory events. Accordingly, in the present study, we attempted to elucidate the inflammatory potential of Tec kinase by investigating the effect of Tec kinase on the inflammatory response induced by LPS in mouse macrophages. To investigate the underlying mechanisms, the involvement of NF-κB and upstream kinase TAK1 was also examined.

## RESULTS

### LPS induces phosphorylation of Tec kinase in RAW264.7 cells

**Figure 1:**
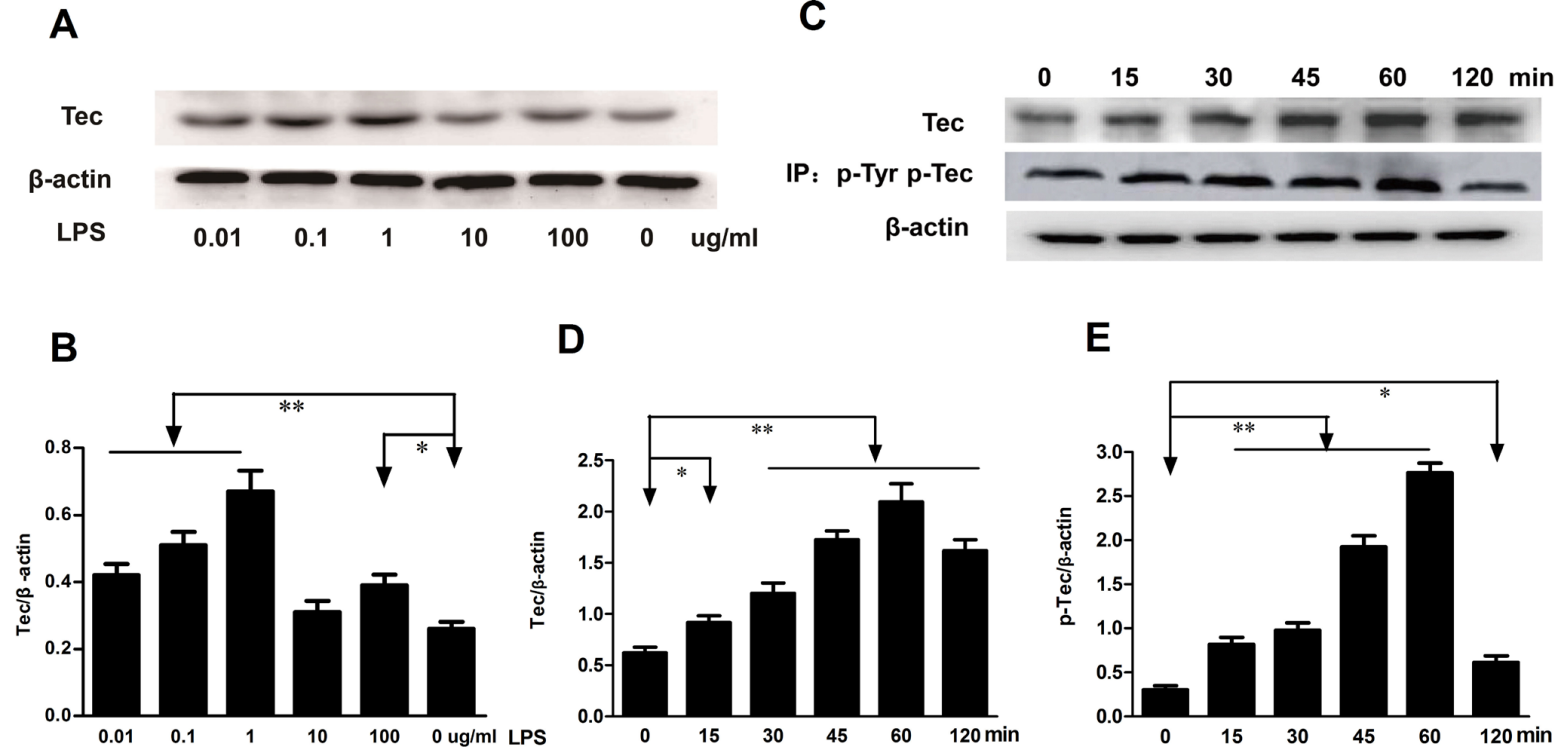
LPS induced the expression of Tec and phosphorylation of Tec protein **A**. The expression of Tec was detected by western blotting after LPS stimulus at different concentration (0.01, 0.1, 1, 10, 100 μg/ml). **B**. Quantitative analysis of Tec protein was determined by integral optical density (*P < 0.05, **P < 0.01, *n* = 3.). **C**. The expression of Tec and p-Tec was detected by Western blotting after 0.1 μg/ml LPS stimulus. Total Tec protein was immunoblotted with an anti-Tec Ab, and tyrosine phosphorylation was detected by immunoprecipitating with an anti-phosphotyrosine Ab. **D**.-**E**. The relative levels of Tec and p-Tec were quantified. 0.1 μg/ml LPS can upregulate the expression of Tec and p-Tec with a peak at 60 min. (*P < 0.05, **P < 0.01, *n* = 3.).

Monocytes and macrophages are vital to the regulation of immune responses and the development of inflammation. We examined whether the Tec kinase was activated after LPS stimulation in macrophages. RAW264.7 cells were incubated with various concentration of LPS (0.01-100 μg/ml) for 24h. The expression of Tec protein exhibited an increasing trend with the alteration of the concentration of LPS. Dose-dependent upregulation of Tec expression was detected by Western blotting assay and represented in Figure 1A. The curve peaked at a concentrate of 1 μg/ml compared with the control group (*P* < 0.01) (Figure 1B). Subsequently, RAW264.7 cells were incubated with 0.1 μg/ml LPS at different indicated times. Immunoblot analysis revealed that LPS increased Tec expression in a time-dependent upregulating manner (Figure 1C), and a peak appeared 60 min after LPS stimulation. To measure Tec kinase activation in RAW264.7 macrophages, tyrosine phosphorylation, correlating with Tec kinase activity was also assessed by immunoprecipitation experiment. Exposure to LPS resulted in an increasing level of Tec at 15min, peak at 60min, (Figure 1C and 1D). Tyrosine phosphorylation was also measured, which correlates with Tec kinase activity. As shown in Figure 1C and 1E, phosphorylated Tec was detected at 15min after LPS stimulation, peak at 60 min, and returns to a higher level by 120 min. These results indicated that Tec kinase activation could potentially participate in the development of inflammation.

### Inhibition of Tec kinase decreases LPS-induced MCP-1 level and mRNA expression in RAW264.7 cells

**Figure 2:**
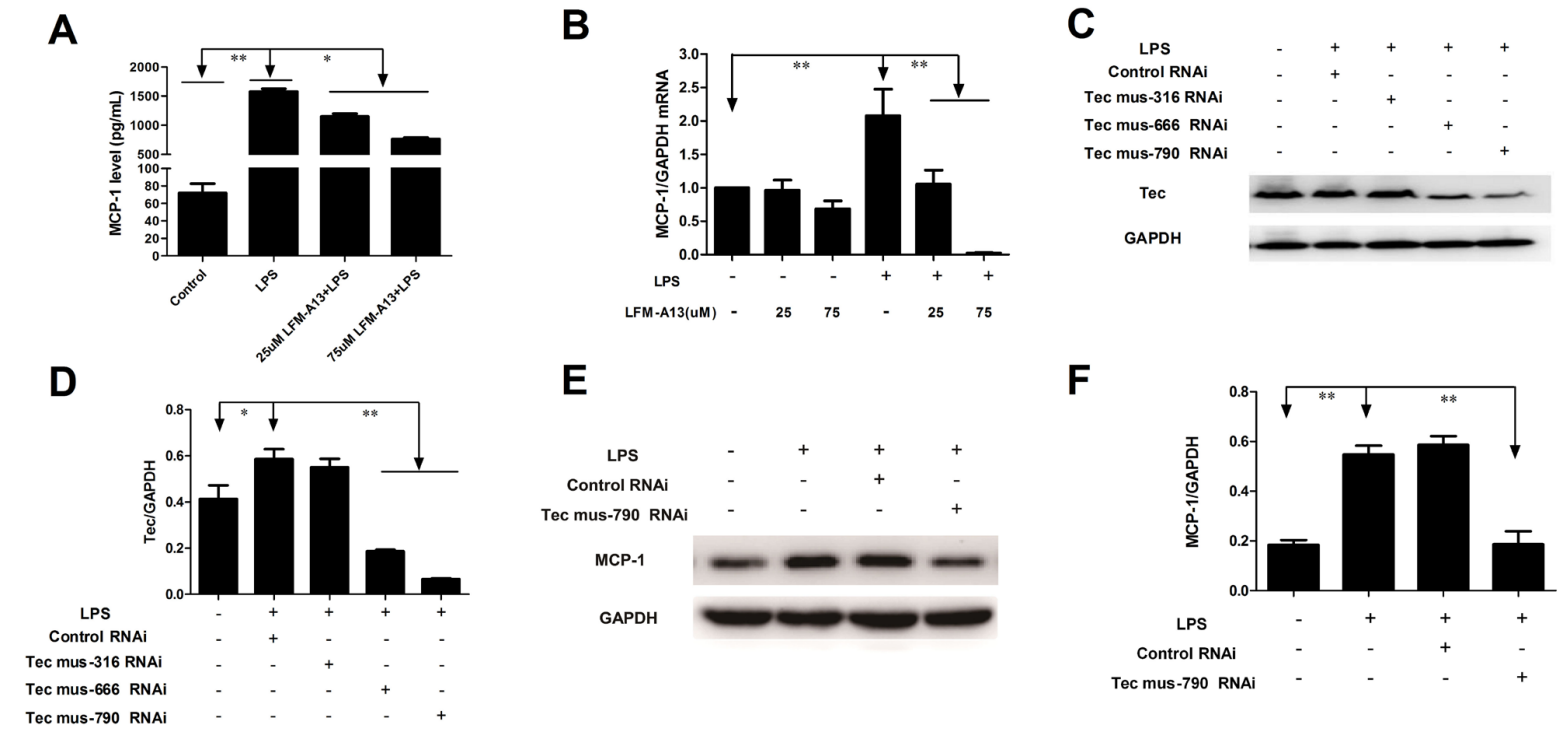
Effects of LFM-A13 or siRNA pretreatment on MCP-1 mRNA expression and protein level in RAW264.7 cells after LPS exposure **A**. RAW246.7 cells were preincubated with LFM-A13 as Materials and Methods. Then the cells were cultured with 0.1 μg/ml LPS for 2 h. Protein level of MCP-1 in supernatants in different group were measured by ELISA. **B**. MCP-1 mRNA in RAW264.7 cells were detected by RT-PCR. LPS stimulus upregulates mRNA and protein level of MCP-1 in RAW264.7 cells, which can be suppressed by LFM-A13. **C**.-**D**. RAW246.7 cells were transferred with Tec-siRNAs or control siRNA as Materials and Methods prior to LPS exposure. Tec mus-666 RNAi and mus-790 RNAi inhibited Tec expression compared with control siRNA, while Tec mus-316 RNAi showed no inhibitory effect. **E**.-**F**. Tec mus-790 RNAi was selected as specific siRNA targeting Tec kinase. Blockage of Tec kinase by Tec mus-790 RNAi significantly suppressed LPS-induced MCP-1 expression. The relative levels were quantified. All data were expressed as means ± SEM. *P < 0.05, **P < 0.01, *n* = 3.

MCP-1 a prototype of CC chemokines, is a potent chemoattractant and a regulatory mediator involved in a variety of inflammatory diseases [2]. The concentrations of MCP-1 in supernatant were determined by ELISA. The level of MCP-1 increased significantly after 0.1μg/ml LPS stimulation for 2 h compared with those in control groups (*P* < 0.05, respectively, Figure 2A). LFM-A13, a leflunomide metabolite analogue, has no effect on the levels of MCP-1 in supernatant. However, preincubation with LFM-A13 (25 μM or 75 μM) prior to LPS stimulation resulted in an obvious decrease in RAW264.7 cell (*P* < 0.05). We then evaluated the effect of LFM-A13 on MCP-1 mRNA expression. The mRNA level of MCP-1 was measured by quantitive real-time PCR. The GAPDH gene expression in all groups was unaffected significantly. Compared with the control group, 0.1 μg/ml LPS stimulus significantly increased the mRNA levels of MCP-1 (*P* < 0.01). As shown in Figure 2B, preincubation with LFM-A13 (25 μM or 75 μM) for 1 h effectively reduced the LPS-medicated MCP-1 mRNA expression.

*In vitro*, we further designed and synthesized Tec kinase siRNA, transfected RAW264.7 cells to verify the role of Tec kinase. We found that Tec mus-666 RNAi and mus-790 RNAi targeting Tec gene reduced the Tec protein level to 3.13 and 8.79 folds in RAW264.7 cells stimulated by LPS respectively, when compared to the control RNAi (*P* < 0.01, Figure 2C and 2D). However, the difference of Tec expression was insignificant between Tec mus-316 RNAi and the negative control siRNA group after LPS exposure. Therefore, Tec mus-790 RNAi was selected as specific RNAi targeting Tec gene. As shown in Figure 2E and 2F, Tec mus-790 RNAi inhibited LPS-induced MCP-1 expression significantly (*P* < 0.01). These results suggest that inhibition of Tec kinase negatively regulated MCP-1 production at the transcription level.

### Inhibition of Tec kinase decreases LPS-evoked ICAM-1 release and mRNA expression in RAW264.7 cells

**Figure 3:**
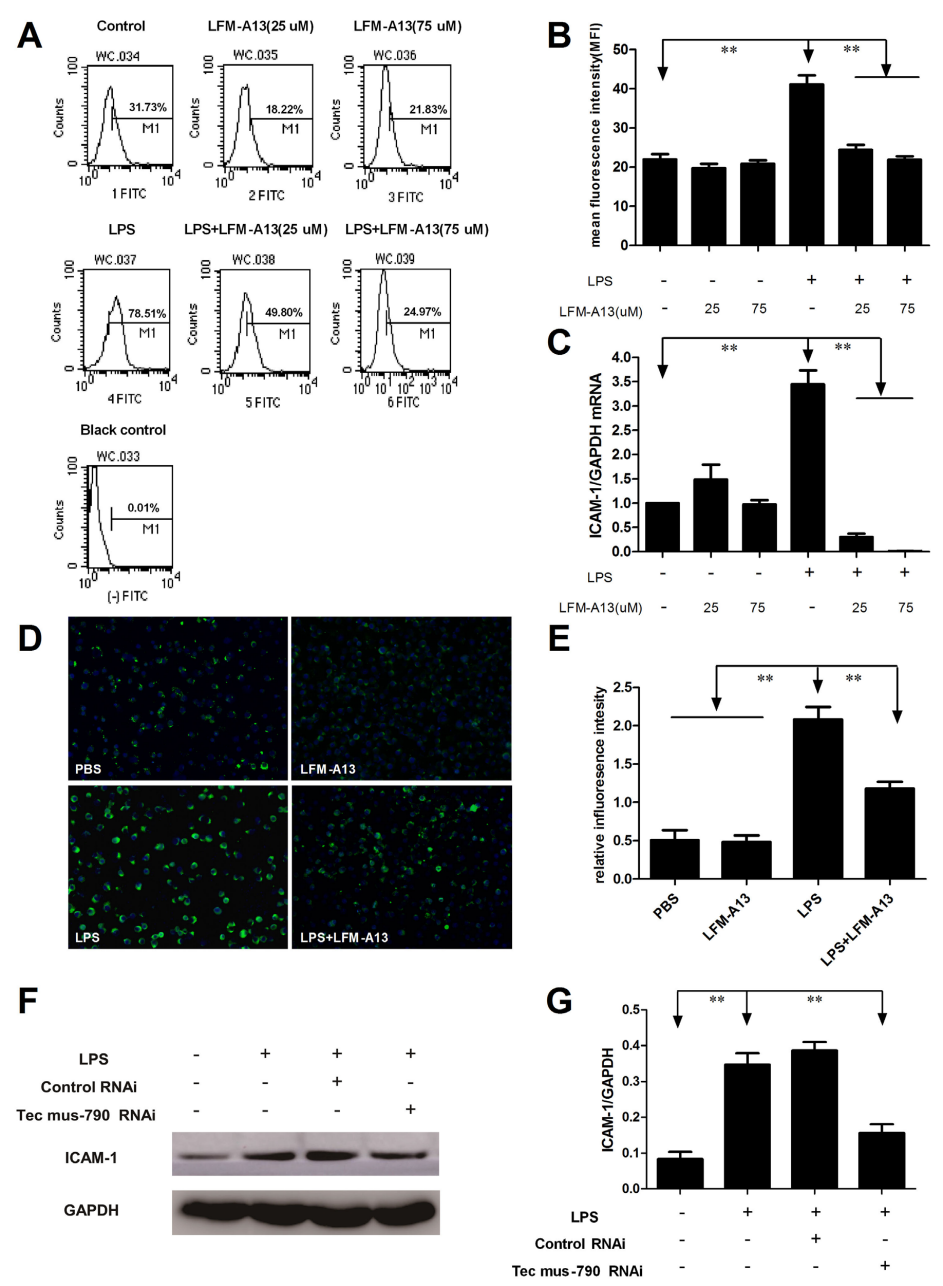
Effect of LFM-A13 or siRNA pretreatment on LPS-induced ICAM-1 expression **A**. The RAW264.7 cells were stimulated by 0.1 μg/ml LPS with/without LFM-A13 preincubtion for 1 h. Intracellular ICAM-1 was detected by flow cytometry as described in Materials and methods. **B**. The relative levels of ICAM-1 were quantified. **C**. ICAM-1 mRNA was assessed by qRT-PCR. **D**. Representative influorescence staining was used to detect the expression of ICAM-1 at 200 × magnification. **E**. The relative influorescence intensity were quantified. **F**.-**G**. Tec mus-790 RNAi inhibited LPS-induced ICAM-1 expression significantly. The relative levels were quantified. All data were expressed as means ± SEM. **P < 0.01, *n* = 3.

ICAM-1, an important intercellular adhesion molecule, has been implicated in inflammatory process and the transmigration of polymorphonuclear neutrophils in sepsis [10]. We therefore examined the expression of ICAM-1 by flow cytometry assay. As shown in Figure 3A and 3B, the expression of ICAM-1 increased markedly in RAW264.7 cells stimulated by LPS (*P* < 0.01). LFM-A13 (25μM or 75μM) significantly inhibited LPS-induced ICAM-1 production in RAW264.7 cells (*P* < 0.01). However, the inhibitory effect of LFM-A13 was not dosage-dependent. Furthermore, ICAM-1 mRNA expression in RAW264.7 cells was investigated to determine whether the LFM-A13 effect was related to its inhibition on mRNA expression of ICAM-1. LFM-A13 (25μM or 75μM) treatment alone showed no significant difference in the level of ICAM-1 mRNA expression and production compared with the control group. On the other hand, the results from the qRT-PCR assays showed that LPS increased ICAM-1 mRNA expression and that LFM-A13 could decrease ICAM-1 mRNA expression induced by LPS (Figure 3C). A similar result was presented by influorescence staining. As showed in Figure 3D and 3E, ICAM-1 expression was found in RAW264.7 cell membrane with green fluorescence. Mean fluorescence intensity of ICAM-1 protein in LPS group was significantly higher than that in control group. However, LFM-A13 obviously decreased green fluorescence intensity in LPS-induced RAW264.7 cell. Moreover, the Tec mus-790 RNAi inhibited LPS-induced ICAM-1 expression significantly (*P* < 0.01, Figure 3F and 3G).

### Inhibition of Tec kinase suppresses LPS-induced activation of NF-ĸB pathway

**Figure 4:**
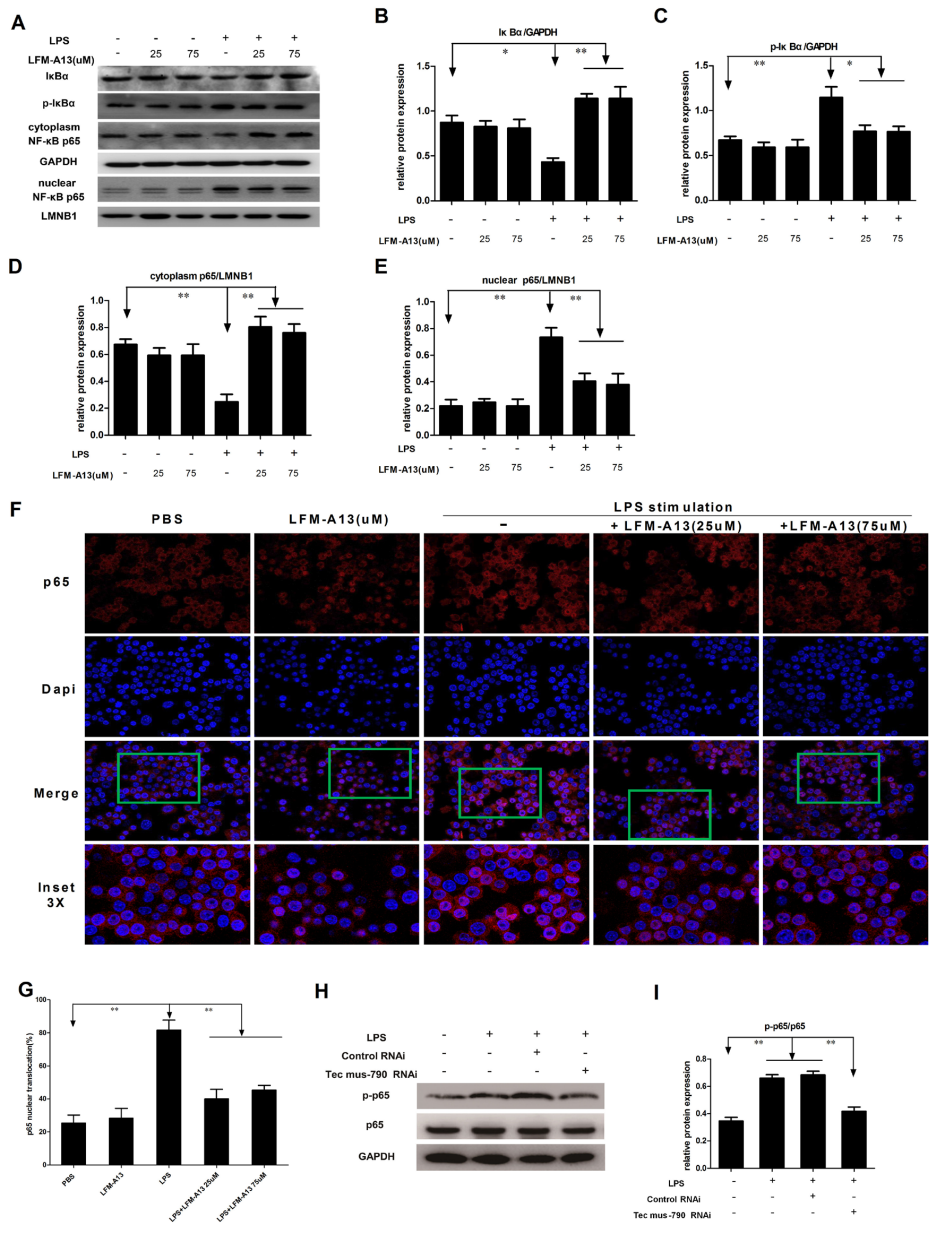
Effect of LFM-A13 or siRNA pretreatment on activity of NF-κB pathway **A**. Expression of IκBα, p-IκBα and NF-κB p65 in cytoplasm and nuclei was analyzed by immunoblotting. **B**.-**E**. The relative expression of IκBα, p-IκBα and p65 in cytoplasm and nuclei were quantified with a densitometry. *P < 0.05, **P < 0.01, *n* = 3. LFM-A13 protected degradation of IκBα in cytoplasm and reduced nuclear accumulation of p65 in RAW264.7 cells exposed to LPS. While, there is no significant difference between the control and LFM-A13 group. **F**. Representative staining of NF-κB p65 localization was observed by using immunofluorescence confocal at a 200 × magnification. Nuclear translocation of p65 was detected by red in RAW264.7 after LPS stimulus with/without LFM-A13 pretreatment. DAPI counterstain identifies cell nuclei. LPS promoted the rapid nuclear localization of p65, whereas LFM-A13 abrogated the translocation of p65 from cytoplasm to nucleus in LPS-stimulated RAW264.7 cells. **G**. Summary of p65 translocation of immunofluorescence confocal. **H**.-**I**. Blockage of Tec kinase by Tec mus-790 RNAi significantly suppressed LPS-induced NF-κB p65 phosphorylation. The relative levels were quantified. All data were expressed as means ± SEM. **P < 0.01, *n* = 3.

Since NF-ĸB is also an important proinflammatory transcription factor, we postulated that Tec kinase may regulate the activation of NF-κB pathway in macrophages. In the canonical NF-κB pathway, NF-κB activation depends on IκBα phosphorylation and degradation and nuclear translocation of p65. We then examined the effect of LFM-A13 on the expression of active NF-κB protein in RAW264.7 cells. As shown in Figure 4A-4C, LPS decreased the levels of IκBα and enhanced IκBα phosphorylation (*P* < 0.05 and *P* < 0.01, respectively). LPS also reduced cytoplasmic NF-κB p65 while elevated nuclear NF-κB p65 (*P* < 0.01, Figure 4A, 4D-4E), indicating that LPS stimulated nuclear translocation of p65 and activated NF-κB signaling. Consistently, LFM-A13 abolished LPS-induced nuclear p65 expression, while increased the levels of IκBα and suppressed IκBα phosphorylation (Figure 4A-4E). On the other hand, LFM-A13 (25μM or 75μM) treatment alone showed no significant difference in the levels of IκBα, phosphoated IκBα and p65 compared with the control group. To further investigate the NF-κB activity, we used indirect immunofluorescence and confocal microscopy to evaluate the nuclear localization of p65 in RAW264.7 cells. As shown in Figure 4F-4G, LPS alone promoted abundant nuclear translocation of p65, whereas LFM-A13 significantly inhibited the translocation of p65 in response to LPS stimulus (*P* < 0.01). These data indicate that Tec kinase, as an upstream protein, involved in LPS-induced activation of NF-ĸB pathway, which in turn mediated cytokines and chemokines production. Moreover, it was also observed that knockdown of Tec by Tec mus-790 RNAi obviously abrogate NF-κB p65 phosphorylation in RAW264.7 cells exposed to LPS (*P* < 0.01, Figure 4H and 4I).

### TAK1 involves in LPS-induced NF-ĸB activation

**Figure 5:**
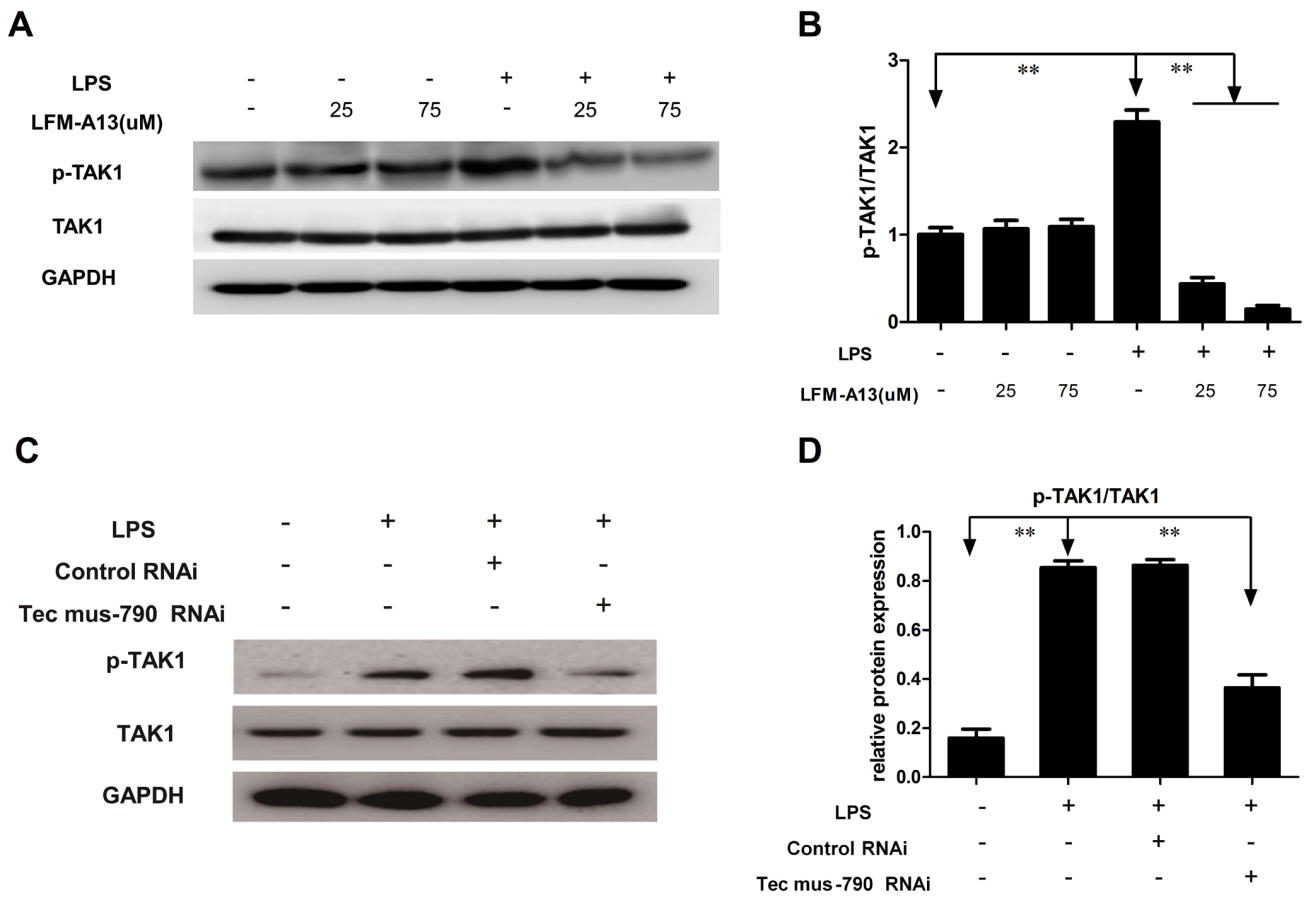
Effect of LFM-A13 or siRNA pretreatment on TAK1 expression **A**. Total TAK1 and its phosphorylation were detected by western bolt. LFM-A13 pretreatment reduced LPS-induced phosphorylated TAK1 expression in RAW246.7 cells, whereas there was no significant for total TAK1 between groups. **B**. The relative levels of p-TAK1/TAK were quantified. **C**.-**D**. Tec mus-790 RNAi-mediated knockdown Tec gene significantly reduced phosphorylated level of TAK1. Densitometric analysis of the results expressed as arbitrary units of mean ± SEM of each group. **P < 0.01, *n* = 3.

Next, we explored the mechanism underling Tec kinase-mediated modulation of NF-kB activity. TAK1, a member of the MAPKKK family, mediates MAPK kinase and NF-κB activation [11]. To address whether TAK1 acted as an upstream regulator of LPS-induced NF-κB activation, TAK1 activity was assessed by Western blotting using anti-p-TAK1 antibody in the present study. As shown in Figure 5A and 5B, higher expression of phosphorylated TAK1 was observed in RAW264.7 cells stimulated by LPS compared to control group (*P* < 0.01). LPS-induced phosphorylation of TAK1 was greatly attenuated in RAW264.7 cells pre-incubated with LFM-A13 in a dosage-dependent manner (*P* < 0.01), indicating that TAK1 activation is required for Tec kinase activity in RAW264.7 cells stimulated with LPS. Furthermore, Tec mus-790 RNAi mediated knockdown Tec gene significantly reduced phosphorylated level of TAK1 (Figure 5C and 5D). These results imply that Tec kinase maybe contributed to cytokines and chemokines productions via NF-ĸB activation dependent on TAK1.

## DISCUSSION

Recently, Tec kinase family such as Tec and Bmx was proved to be trapped in chronic inflammatory diseases such as gouty and rheumatoid arthritis [12, 13]. Following various stimulus, Tec family kinases are recruited to the plasma membrane through binging of their pleckstrin homology (PH) domain to phosphatidylinositol(3,4,5)-trisphosphated (PIP3) [14]. Then they are phosphorylated within their activation loop by either autophosphorylation or by Scr-family kinases [15, 16]. Zen et al [17] has implicated that chemokine fMLP stimulated human neutrophil induced membrane translocation and phosphorylation of BMX, Btk and Tec kinases. Src family kinase inhibitor PP1 and PP2 significantly inhibited the adhesion and migration ability of neutrophil. In Zemans's study [18], direct evidence that an increase in the phosphorylation of both Tec and Btk in human neutrophils exposed to LPS was obtained. Tec kinase was also activated with the translocation to the plasma membrane. Similar findings were noted in a study of Zwolanek et al [19] investigating that Syk-dependent Tec kinase was required for inflammatory response in murine peritoneal macrophages invaded by the human fungal pathogen C. albicans. Tec family kinases such as Tec and Btk are essential for M-CSF (M-CSF)-induced signaling pathways that regulate macrophage survival [20]. In present study, Tec kinase was induced and activated as phosphorylation in LPS-exposed RAW264.7 cells. The results suggest that Tec kinase perhaps is an important mediator of the inflammatory immune response in macrophages.

Macrophages play a critical role in activating inflammatory process in the cellular response to endotoxin. Wang et al [21] has shown that Tec kinase as a mediator enhance IL-8 transcription in RAW264.7 cells simulated with LPS. Point inactivating mutations in SH2, PH or PTK domain almost completely abolish the effects of Tec kinase on inflammation *in vitro*. In Tampella’ s study [22], they have also demonstrated that primary resident peritoneal macrophages deficient for Btk and Tec kinases secreted less proinflammatory cytokines such as IL-6, IL-12 and TNF-α in response to TLR agonist (LPS and Pam3CSK4) stimulation than do wild-type cells. Palmer et al. [23] generated Btk^−/−^ knock-out human macrophages. They found suppression of Btk expression resulted in a significant decrease in LPS-induced TNF expression, with IL-6 production remaining unchanged. Thus, the involvement of Tec kinase in TLR-induced cytokine production in human and murine monocytes remains controversial. MCP-1 is one of the key chemokines that regulate migration and infiltration of monocytes/macrophages. In current study, it showed that MCP-1 was overexpressed in macrophages incubated by LPS. The increasing expression of gene also was presented.

We next used a pharmacologic inhibitor, LFM-A13 and as well as siRNA silencing of the expression of Tec to investigate the functional responsiveness in macrophage. LFM-A13 is a selective Tec family kinase inhibitor. It has been recently identified as Btk/Tec-kinase inhibitor [18, 24, 25], and shows a potent inhibitor of Polo-like kinase (IC50 = 10 μM) [26, 27]. Existing evidence suggested that LFM-A13 reduces cytokines production in neutrophils at different concentration (25 uM 50 uM, or even 100 uM) and inhibits the activation of three of the Tec kinase such as Tec, Btk, Bmx [9, 13, 18]. Therefore, there is some controversy on LFM-A13. These events need further studies to certify. In this study, LFM-A13 decreased mRNA expression and MCP-1 production in supernatants of LPS-stimulated macrophage at 25 or 75 uM. Tec siRNA also inhibited MCP-1 expression. LFM-A13 decreased MCP-1 RNA expression by 90% but only decreased MCP-1 protein levels by 30%. It is not clear whether regulation of translation is involved in these significant differences between gene expression and protein levels. It also needs further study. Although LFM-A13 is not the best reagent for specific targeting Tec kinase, the combined results of siRNA suggest that Tec mediated-overexpression of cytokines perhaps contributes to the systemic inflammatory response in LPS challenge.

ICAM-1, a transmembrane protein, is a member of the immunoglobulin superfamily. It is presented constitutively on the surface of endothelium and other cells including lymphocytes and monocytes/macrophages [28, 29]. The primary function of ICAM-1 is to stabilize cell-cell interactions and facilitate leukocyte endothelial transmigration. Many previous studies have confirmed that ICAM-1 can be induced and by TNF-α, IL-1 and other pathogens [30, 31]. When activated, interaction of ICAM-1 with β2-integrins enhances monocytes binging to endothelium tightly and promotes leukocyte aggregation, infiltration and extravasation [32, 33]. In this study, LPS increased the expression of ICAM-1 mRNA and protein level in macrophages. The current study further demonstrated that the highest density was seen in LPS-stimulated RAW264.7 cells by flow cytometry and Immunofluorescence stain. Since ICAM-1 is identified as an important mediator in such setting, it is therefore to observe and quantify whether there have been any perturbation in RAW264.7 cells pretreated with LFM-A13. Consistent with MCP-1, LFM-A13 attenuated ICAM-1 mRNA and protein levels and weakened its fluorescence density in macrophages. Tec siRNA also decreased ICAM-1 expression induced by LPS. Taken together, the current findings suggest that Tec kinase may be involved in regulation of ICAM-1 productions in macrophages, further infer that Tec kinase could act as a potential mediator, triggering the inflammatory cascade in LPS challenge.

Our previous evidence and literature indicates that a key signaling molecules namely, NF-κB plays a crucial role in acute inflammatory response [34–36]. Its target genes regulating inflammation include MCP-1 and ICAM-1 [34, 37, 38]. More solid evidence tying NF-κB activation to cytokine cascade was just provided in this study. Employing LFM-A13 or knock-down of Tec kinase downregulated phosphorylated NF-κB p65 induced by LPS *in vitro*. Moreover, we further identified the positive correlation between ugregulation of Tec kinase and activation of NF-κB. The experiment showed that LPS enhanced nuclear translocation of p65 via phosphorylation and subsequent degradation of IκBα, indicating the activation of NF-κB pathway. Tec kinase inhibitor can significantly block this effect. In Yu's study [39], NF-κB subunit p65/RelA was identified and directly induced transcriptional activity of the Tec gene promoter. Moreover, they also found that proteasome inhibitors repressed Tec transcription through inactivation of the NF-κB signaling pathway. Altogether, this evidence strongly suggests that the inflammatory activation of Tec kinase in macrophages stimulated by LPS correlated with activation of NF-κB pathway.

TAK1, a member of MAPK family, was originally identified as a kinase in activating pro-inflammatory signals [11, 40]. Accumulating evidence has indicated that the phosphorylation of TAK1 is involved in activation of MAPKs and NF-κB signal pathway [41–44]. These pathways triggered by various stimuli converge at the level of TAK1, and subsequently leads to expression of proinflammatory mediators. In the rat model of septic shock [45], LPS-induced hypotension, tachycardia, and inflammation was associated with increased MyD88 expression and phosphorylation of TAK1 and activation of NF-κB. In the Shao's study [46], blocking of NF-κB activation by 7b, an anti-inflammatory agent, was mediated by inhibiting TAK1-downstream ERK1/2 and p38MAPK in LPS stimulated RAW264.7 cells and primary mouse macrophages. Gottar-Guillier et al. [47] has implicated that Bmx is involved in rheumatoid arthritis. Transient depletion of BMX strongly reduced secretion of IL-8 *in vivo* and *in vitro*, inhibiting phosphorylation of p38 MAPK and JNK, as well as activation of NF-κB dependent on TAK1. This notion was supported by the current study that phosphorylation of TAK1 was abolished with LFM-A13 treatment or Tec-siRNA transference in LPS-stimulated RAW264.7 cells. The results suggest that inhibitory modulation of TAK1 is an upstream event required for the anti-inflammatory activity of LFM-A13 or Tec-siRNA. Taken together, these findings also provided evidence that Tec kinase interact with the adaptor molecule TAK1 regulating downstream NF-κB.

In summary, the results of our experiments indicate that LFM-A13 inhibited the LPS-induced increase of MCP-1 and ICAM-1 at mRNA and protein levels in RAW264.7 cells. Additionally, LFM-A13 or Tec-siRNA abrogated the activation of TAK1/NF-κB pathways. Our data clearly demonstrate that Tec kinase acts as a shared and essential component of inflammatory signaling pathway, providing especially attractive therapeutic target for LPS challenge.

## MATERIALS AND METHODS

### Cell culture and reagents

Mouse macrophage cell line RAW 264.7 cells were obtained from the Chinese Type Culture Collection(Shanghai Institute of Cell Biology, China) and grown in the Dulbecco Modified Eagle Medium (DMEM, GiBCO), supplemented with 10% fetal bovine serum (FBS, GIBCO), 100 U/mL penicillin, 100U/mL streptomycin at 37°C in a humidified atmosphere of 5% CO2. Cells were seeded between passages 3-5 (1 × 10^5^ cells/well) on 6-well BioFlex collagen-coated culture plates and grown to 80% confluence. FBS concentration was reduced to 1% 24 hours prior to experiments in order to avoid interference from its components and to synchronize the cell cycle. The cells were stimulated with LPS (Sigma, St. Louis, MO) by different concentrations and time courses. To study the role of Tec kinase in LPS-induced cytokine releases in macrophages. LFM-A13, a leflunomide metabolite analogue, at different concentrations was added to the cells at 1 h before stimulation with LPS.

Reagents used in this study were purchased from the following sources: LFM-A13 from Tocris Bioscience; Tec siRNA from Genepharma Co, Ltd (Shanghai, China); All monoclonal antibodies for NF-κB p-p65, p65, IκBα, p-IκBα, were purchased from Protein Tech Group (Chicago, IL, USA). Anti-β-actin, LMNB1, GAPDH, TAK1 and p-TAK1 were obtained from Cell signaling Technology (Danvers, MA, USA). Antibodies for Tec protein and p-Tyr (4G10) were obtained from Santa Cruz Biotechnology (Santa Cruz, CA).

### Enzyme-linked immunosorbent assay

The MCP-1 level was measured in supernatants using a commercial enzyme-linked immunosorbent assay (ELISA) with kit (R&D Systems Europe, Abingdon, United Kingdom), according to the manufacturer's instructions. All samples were run in duplicate and averaged.

### Flow cytometry

ICAM-1 expression was measured as previously described [48]. Briefly, RAW264.7 cells were cultured on 6-well plates until confluent and pretreated with LFM-A13 (25 μM or 75 μM) for 1 h before LPS exposure. After the treatment, cells were washed with cold PBS and incubated with 5 μM CM-H2DCFDA for 30 min, followed by several additional washes with cold PBS. For FACS analysis, cells were removed from coverslips with trypsin+EDTA/HBSS, incubated with anti-mouse ICAM-1 (CD54) antibody (R&D Systems) or a normal mouse isotype-matched IgG control (R&D Systems) followed by an anti-mouse IgG-FITC secondary antibody (Sigma-Aldrich), rinsed in PBS, fixed in a 2% paraformaldehyde/PBS solution and analyzed using a flow cytometer (FACScalibur, BD Biosciences, San Jose, CA) at an emission wavelength of 525 nm. ICAM-1 expression was analyzed using FlowJo software (Tree Star, Inc).

### Immunofluorescence staining and confocal microscopy

RAW264.7 Cells were seeded and cultured in 12-mm glass coverslips. The cells were fixed in 4% paraformaldehyde for 15 min at room temperature, then permeabilizd with 0.25% X-Triton100 for 20 min. After washed with PBS three times, cells were blocked with 3% bovine serum albumin in PBS for 1h followed by incubation with diluted 1:100 rabbit anti-ICAM-1 (R&D Systems) or 1:100 rabbit polyclonal anti-p65 at 4 °C overnight. After thoroughly washed with PBS, fluorescein isothiocyanate (FITC)-conjugated goat anti-rabbit IgG (Santa Cruz, CA) or labeled with Alexa Flour 555 was applied for 1h in the dark. Cells were then incubated with DAPI (4’,6-diamidino-2 phenylindole) for nuclear staining for 5 min. Fluoromount G was used as mounting medium. DAPI-associated fluorescent staining and ICAM-1 antibody-associated green staining were detected by fluorescent microscopy. Images of p65 stain were acquired by Leica SP5 (Carl Zeiss, Oberkochen, Germany) confocal microscope at a 200 × magnification.

### Preparation of cytoplasmic and nuclear extracts

Cultured macrophages (5-10^6^) were washed twice with PBS, lysed and scraped off the dishes, transferred to a 1.5-mL tube, and centrifuged. The cytoplasmic and nuclear protein extracts from RAW264.7 cells were prepared as described in our previous study [38]. In brief, Cells were harvested and lysed with hypotonic buffer (10 mM HEPES, 10 mM KCl, 0.1 mM MgCl2, Triton X-100 and 20% glycerol, pH 7.9) on ice for 5 min and centrifuged for 5 min at 5000 rpm. The supernatant was treated as cytoplasmic fraction. The pellet was then lysed with hypertonic buffer (10 mM HEPES, 400 mM NaCl, 0.1 mM MgCl2, 0.1 mM EDTA, Triton X-100 and 20% glycerol, pH 7.9) on ice for 20 min. After centrifugation at 15000 rpm for 5 min, supernatants were used as nuclear fractions. Protein concentrations were determined using the bicinchoninic acid (BCA) protein assay reagent (Pierce Chemical Company, Rockford, IL, USA), according to the manufacturer's instruction.

### Western blotting and immunoprecipitation analysis

SDS-PAGE and Western blotting were performed according to standard procedures. Briefly, cytoplasmic or nuclear protein samples were subjected to electrophoresis on 4-12% SDS-polyacrylamide gradient gels and transferred to a PVDF membrane. After overnight blocking with 2% BSA in TBS-Tween 0.1%, the membranes were then immunoblotted with primary antibody for Tec (1:1000), NF-κB p65 (1:1000), phosphorylated p65 (1:1000), IκBα (1:500), phosphorylated IκBα (1:500), GAPDH (1:1000), LMNB1 (1:1000), TAK1 (1:1000) or phosphorylated TAK1(1:500) antibodies overnight at 4°C. Anti-rabbit HPR conjugated antibody was used as a secondary antibody followed by enhanced chemiluminescence detection system (Amersham, Little Chalfont, UK). All Western blotting were repeated three times.

The immunoprecipitations (IP) were performed as described previously [49]. In brief, cell lysates supernatants were pre-cleaned with protein A agarose beads (Santa Cruz) for 1 h at 4°C. The clear lysates were incubated with anti-Tec antibody (diluted 1:100) for overnight at 4°C. Immune complexes were captured on beads and washed three times in whole-cell extract buffer, and precipitated protein were eluted and separated by SDS-PAGE. After trarsmembrane, immunoblotting for p-Tyr (diluted 1:100) was performed.

### RNA extraction and quantitative real-time PCR (qRT-PCR)

Total RNA extraction was performed using Trizol reagent (Invitrogen Life Technologies, USA) according to manufacturer's protocol. RNA from each culture was treated with DNase to remove any contaminating genomic DNA. The quality of RNA was assessed by 1% agarose gel electrophoresis and was quantitated by NanoDrop ND-1000 (Thermo Fisher Scientific, Wilmington, DE, USA) spectrophotometer.

Subsequently, 2μg of Total RNA was reverse transcribed into cDNA using a Reverse Transcriptase System (Promega, Madison, USA). qRT-PCR was performed using Applied Biosystems 7500 Fast Real-Time PCR System with 1 mM primers according to the manufacturer's instructions. Sequences of primers used for qRT-PCR were listed as: MCP-1: forward 5’-TTGACCCGTAAATCTGAAGCTAAT-3’, reverse 5’-TCACAGTCCGAGTCACACTAGT TCAC-3’; ICAM-1: forward 5’-GGCTGAAAGATGAGCTCGAGAG-3’, reverse 5’- TTCTCAAAGCACAGCGGACTG-3’. Additionally GAPDH (forward 5’-AGCGAGACCC CACTAACA -3’, reverse 5’-GGGGCTAAGCAGTTGGTG -3’) was used as an endogenous reference gene for the normalization. All PCR efficiency values were determined using LinReg. Data was presented as the mean ΔCt score ± standard error of the means. All qRT-PCR reactions were performed in triplicate.

### siRNA interference

To down-regulate Tec kinase expression, three specific siRNA duplexes targeting mouse Tec, described as Tec-mus-316 RNAi (sense: 5’-GUCGAGCGGAGAAGAAAUATT-3’; antisense: 5’-UAUUUCUUCUCCGCUCGACTT-3’), Tec-mus-666 RNAi (sense: 5’-GCGCCAGAAAUAAAGAAGATT-3’; antisense: 5’-UCUUCUUUAUUUCUGGCGCTT-3), Tec-mus-790 RNAi (sense: 5’-GAGGCCAAGAGUAUAUCAUTT-3’; antisense: 5’-AUGAUAUACUCUCGGCCUCTT-3’), were synthesized and obtained from GenePharma Co. Ltd (Shanghai, China). SiRNA duplexes with non-specific sequences were used as negative control. The siRNA transfection into RAW264.7 cells was conducted by Lipofectamine 2000 (Carlsbad, CA) according to the manufacturer's instructions. The knockdown efficiency of specific siRNAs was detected by Western blot assays.

### Statistical analysis

All data were presented as means ± standard error of the mean (SEM) and analyzed by one-way analysis of variance (ANOVA), followed by Student-Newman-Keuls test. All the statistical analyses were performed using GraphPad Prism version 5.0 for Windows (GraphPad Software, San Diego, CA, USA), and statistical significance was set at *P < 0.05; **P < 0.01.
